# The impact of bariatric surgery on gut microbiota: a bibliometric analysis of research trends and scientific contributions

**DOI:** 10.3389/fmicb.2025.1523809

**Published:** 2025-01-29

**Authors:** Shuaichang Gong, Xiabiao Zhang, Xiaoliang Chen, Ping Wan, Longfei Zhou, Jun Zhang

**Affiliations:** ^1^Jiangxi Provincial People's Hospital, The First Affiliated Hospital of Nanchang Medical College, Jiangxi, China; ^2^Jiangxi Medical College, Nanchang University, Jiangxi, China

**Keywords:** obesity, bariatric surgery, gut microbiota, bibliometric analysis, metabolic syndrome

## Abstract

**Background:**

Obesity is a prevalent global disease closely linked to various chronic conditions. Bariatric surgery (BS) is currently recognized as the most effective treatment. Increasing evidence suggests that BS alters the gut microbiota (GM), which plays a crucial role in postoperative weight loss. However, there has been no systematic bibliometric analysis exploring the relationship between BS and GM to date.

**Methods:**

We conducted a literature search on BS and GM from January 1, 1981, to May 25, 2024, within the Web of Science Core Collection (WoSCC). We utilized Excel 2021, VOSviewer 1.6.19, CiteSpace 6.2.R3, and the R package “bibliometrix” 4.3.0, along with the online bibliometric analysis platform (https://bibliometric.com/app), to visualize publishing trends and research hotspots in this field.

**Results:**

A total of 2,542 articles meeting the criteria were included. Since 2015, the rate of publication has significantly accelerated. The United States leads in both the number of publications and average citations per article. The University of São Paulo is the most active institution, whereas the University of Copenhagen has the highest average citation count. Obesity Surgery is the journal with the highest number of publications, and the most prolific author is Karine Clement. Keyword and thematic analyses indicate that “gut microbiota” and “bariatric surgery” are the primary research hotspots for future studies.

**Conclusion:**

In summary, this field is garnering increasing attention. Our findings suggest that future research will likely focus on the effects of bariatric surgery on gut microbiota and its biological mechanisms, the role of gut microbiota in the weight loss process, and the development of combined treatments based on gut microbiota.

## 1 Introduction

Obesity has evolved into a global health crisis. Over the past three decades, the global prevalence of obesity has exponentially increased, with adult obesity rates more than doubling, reaching epidemic proportions. According to statistics from the World Health Organization (WHO) in 2022, there are 2.5 billion overweight adults (aged 18 and above) globally, of which 890 million suffer from obesity (Noubiap et al., 2022). Obesity is closely linked to a variety of severe health complications, including cardiovascular diseases, type 2 diabetes, non-alcoholic fatty liver disease, sleep apnea, osteoarthritis, gastroesophageal reflux disease, and certain types of cancer (Li et al., 2023; Xie et al., 2024). For instance, obesity exacerbates cardiovascular conditions by increasing blood volume and cardiac workload, inducing hypertension, promoting arteriosclerosis, triggering insulin resistance and diabetes, and leading to fatty liver and metabolic syndrome (Powell-Wiley et al., 2021). Additionally, individuals with obesity often face social discrimination and prejudice, resulting in psychological health issues such as depression and anxiety, further burdening the public health system (Westbury et al., 2023).

Treatment options for obesity primarily include lifestyle modifications, pharmacological interventions, psychological therapies, and surgical procedures. Among these, surgical interventions are considered the most effective and sustainable method for weight reduction and also significantly alleviate obesity-related complications (Welbourn et al., 2019; Ming et al., 2021). Common types of weight reduction surgeries include sleeve gastrectomy, Roux-en-Y gastric bypass, single anastomosis gastric bypass, and biliopancreatic diversion with duodenal switch. These procedures achieve weight loss by altering the gastrointestinal anatomy to limit food intake and absorption. Moreover, bariatric surgery also regulates gastrointestinal hormone secretion, such as increasing glucagon-like peptide-1 (GLP-1) levels and reducing ghrelin levels, thereby suppressing appetite and enhancing satiety, which further facilitates weight loss (Luo and Tavakkoli, 2021). Recent studies have found that bariatric surgery significantly impacts the composition and diversity of the gut microbiota, a key factor in sustained postoperative weight loss (Zhang et al., 2024).

The human gut harbors trillions of bacteria, forming a crucial component of the gut microbiome ecosystem and playing a vital role in physiological functions (Sender et al., 2016; Dimidi et al., 2019). With advancements in 16S rRNA sequencing and metagenomic sequencing, extensive research has demonstrated that the gut microbiota is intricately linked with obesity, diabetes, cardiovascular diseases, neuropsychiatric disorders, irritable bowel syndrome, and cancers (Di Tommaso et al., 2021; Jacobs et al., 2023; Larsen et al., 2010; El Tekle and Garrett, 2023; Borkent et al., 2022). Notably, recent findings suggest that changes in the gut microbiota following bariatric surgery may exert long-term effects on weight management and metabolic regulation through mechanisms involving the gut-brain axis, metabolic product formation, and immune modulation, thus being critical factors in weight reduction outcomes (Tremaroli et al., 2015; Liou et al., 2013; Fouladi et al., 2019). Therefore, it is of significant importance to conduct an in-depth study on the role of gut microbiota in weight loss treatments.

Bibliometrics, a technique that applies mathematical and statistical principles for comprehensive analysis of academic literature, was initially proposed by Alan Pritchard in 1969 (Khalil and Gotway Crawford, 2015). Compared to traditional systematic review methods, bibliometrics offers multi-level and multi-faceted analytical capabilities. It allows for quantitative analysis of vast quantities of academic literature, revealing citation relationships and the impact within the field. This analysis not only aids researchers in understanding the overall research dynamics of a particular area but also identifies key research nodes and influential studies. Furthermore, bibliometrics facilitates co-occurrence analysis to uncover relationships and trends among research topics, aiding in the understanding of knowledge evolution and expansion (Ninkov et al., 2022). Additionally, visualization techniques in bibliometrics, such as scientific knowledge maps, co-word analysis graphs, and citation network diagrams, render complex academic data more comprehensible. These maps and networks not only display hotspots and trends in research topics but also assist researchers in discovering potential research directions and collaborative opportunities. For example, by analyzing high-frequency keywords and their trends within a field, researchers can predict future research hotspots and accordingly adjust their research directions and strategies (Thompson and Walker, 2015).

In recent years, an increasing number of clinicians and researchers have focused on studies related to the gut microbiota and bariatric surgery treatment. However, there is currently a lack of systematic bibliometric analysis in this field. To address this gap, this study employs bibliometric methods to systematically visualize literature published from 1981 to 2024 concerning bariatric surgery and the gut microbiota. The research aims to provide new perspectives and ideas for obesity treatment. Specific analytical objectives include: (1) revealing the global development trends of bariatric surgery and gut microbiota research from 1981 to 2024; (2) identifying the most influential countries, regions, institutions, authors, and journals within this field; and (3) elucidating the main focus points and trends in the research on bariatric surgery and gut microbiota, predicting future research hotspots, and offering references for subsequent studies.

## 2 Methods

### 2.1 Data source

The data for this study were obtained from the Web of Science Core Collection (WoSCC) database, accessible at: https://webofscience.clarivate.cn/wos/woscc/basic-search. The WoSCC database was selected because it includes a vast number of authoritative and high-quality journals worldwide, and it provides extensive citation data. This makes WoSCC widely recognized as the most authoritative and comprehensive bibliometric analysis tool.

### 2.2 Search strategy

The search strategy is as follows (January 1, 1981, to May 25, 2024):

#1 TS = microbiota* OR microbiome* OR flora* OR microflora* OR bacteria* OR prebiotic* OR probiotic* OR antibiotic* OR dysbiosis* OR Saccharomyces* OR Lactobacillus* OR Bifidobacterium* OR Escherichia coli*.

#2 TS = Bariatric Surgery OR Obesity Surgery OR Metabolic Surgery OR Weight Loss Surgery OR Bariatric Surgical Procedures OR Sleeve Gastrectomy OR Gastric Banding OR Roux-en-Y Gastric Bypass OR One Anastomosis Gastric Bypass OR Omega Loop Gastric Bypass OR Mini-gastric Bypass OR Single Anastomosis Gastric Bypass OR Single Anastomosis Duodeno-Ileal Switch OR Biliopancreatic Diversion with Duodenal Switch OR Gastric banding OR Adjustable Gastric Banding OR Sleeve Plus Procedures OR Transit Bipartition Surgery OR Sleeve Gastrectomy With Transit Bipartition OR Gastric Balloon OR Jejunoileal Bypass OR Gastric Plication Surgery OR Laparoscopic Gastric Greater Curve Plication.

#3 #1 AND #2 Two researchers independently conducted searches and screenings on the same day. In cases of disagreement, discussions and consultations were held until a consensus was reached. The inclusion and exclusion criteria for this study are as follows (Figure 1):

1. The literature search range is limited to January 1, 1981, to May 25, 2024.
2. Only articles and reviews are included.
3. Only literature published in English is included.

**Figure 1:**
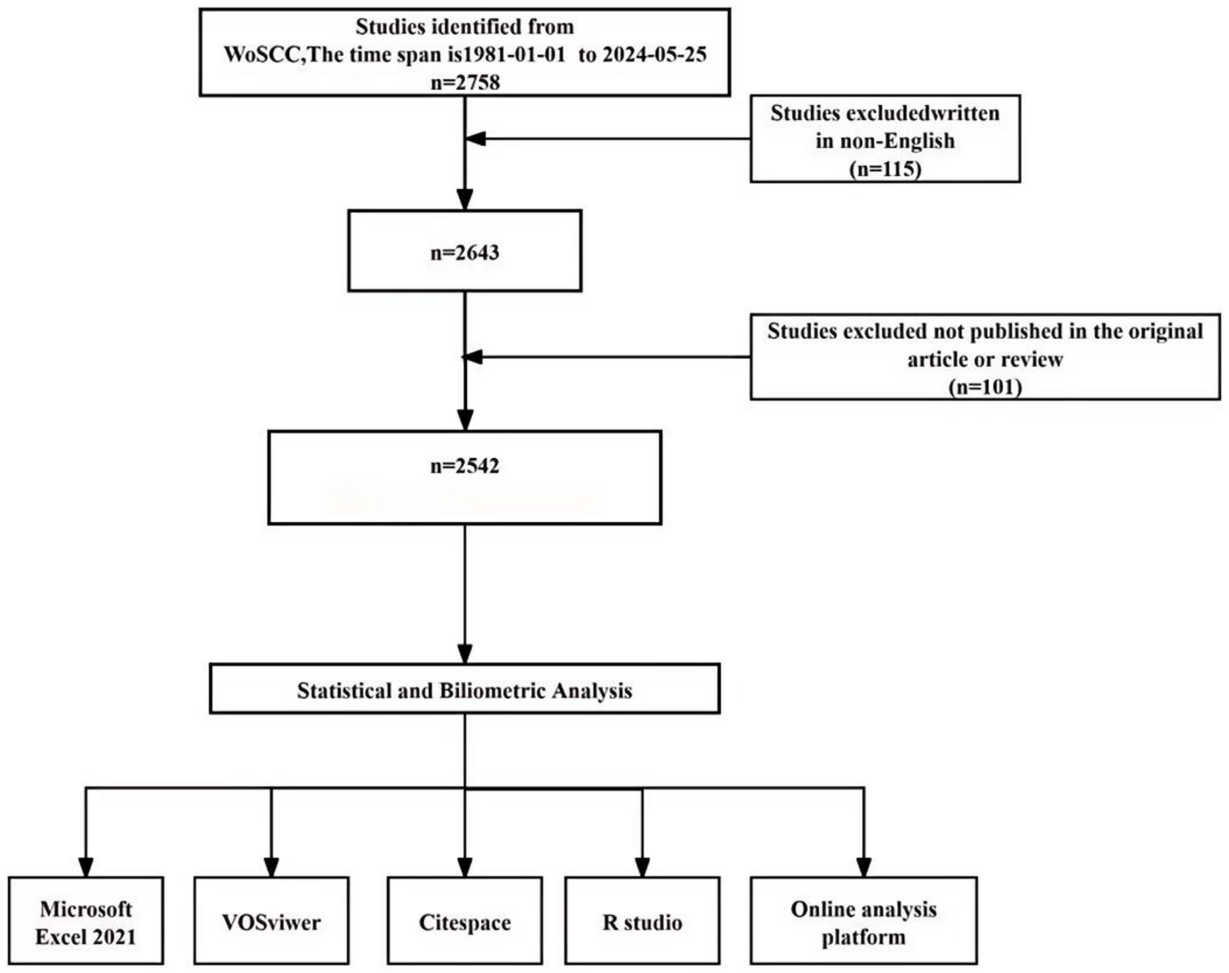
Flowchart of the literature searching and screening in the study.

### 2.3 Data collection

A total of 2,758 documents were retrieved from the WoSCC database. Based on the inclusion and exclusion criteria, 115 non-English documents and 101 non-article or non-review documents were excluded. Consequently, 2,542 documents met the criteria and were downloaded in the form of “full records and cited references” for analysis using bibliometric analysis tools.

### 2.4 Data analysis

To achieve a comprehensive data evaluation, five tools were utilized for bibliometric and visualization analyses. These tools included Microsoft Excel 2021, VOSviewer 1.6.19, CiteSpace 6.2.R3, the “bibliometrix” package in R 4.3.0, and an online analysis platform.^1^ Specifically, Microsoft Excel 2021 was used to generate the research flowchart, integrate data from scientific network databases, and visualize the top 10 funding agencies and the top 5 authors in terms of publication count and h-index. The online analysis platform was employed to create a geographical distribution map of national publications. VOSviewer was used to analyze countries, institutions, authors, and key sources, with the size of the points representing the number of documents and the width of connecting lines indicating the level of collaboration between countries.

## 3 Results

### 3.1 Evolution and development of the literature

**Figure 2:**
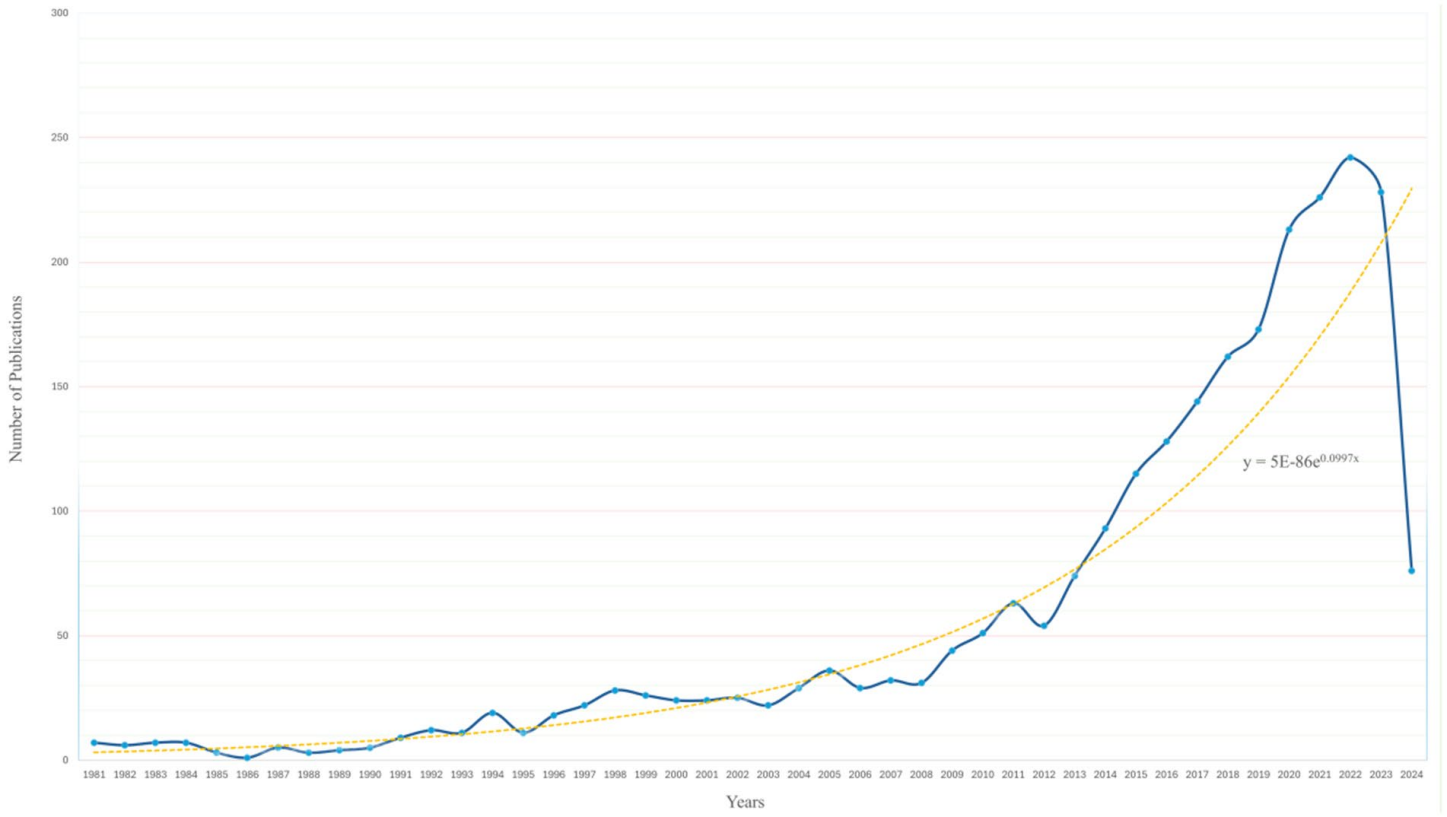
Growth trends of published articles on the BS/GM from January 1, 1981 to May 25, 2024.

Through a systematic literature search, 2,542 documents meeting the inclusion criteria were identified. The earliest publications on gut microbiota and bariatric surgery appeared in 1981. Since then, the number of publications has increased annually, rising from 7 publications in 1981 to an annual average of over 200 publications in the past 4 years. It is noteworthy that the publication count for 2024 is below the average of the last 4 years, likely due to the search cut-off date being in May 2024. The specific trend is illustrated in Figure 2. The growth in publication volume can be roughly divided into three phases: Phase 1 (1981–1991): The number of publications was low and relatively stable. Phase 2 (1992–2014): The publication rate gradually accelerated. Phase 3 (2015–2024): The publication rate significantly increased. This trend reflects the growing interest of researchers in this field, highlighting future research hotspots in the area of weight loss treatments.

### 3.2 Active countries and regions

**Figure 3:**
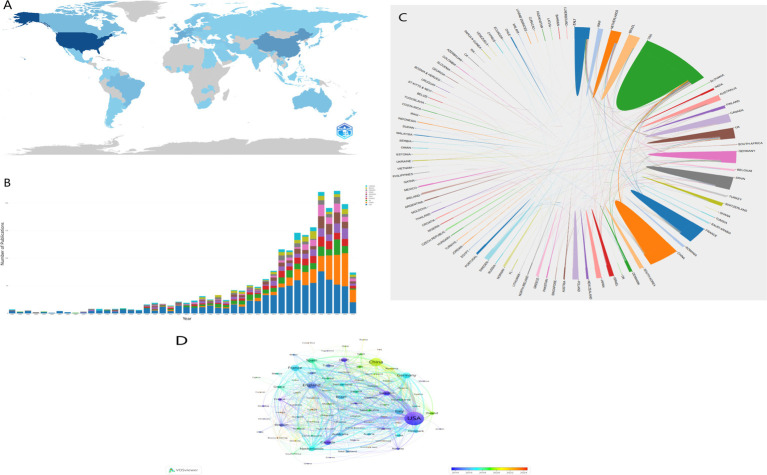
**(A)** The geographic distribution map is based on the total literatures of different countries/regions. **(B)** The annual literatures of the top 10 countries/ regions from January 1, 1981 to May 25, 2024. **(C)** Total number of national communications and cooperation links. **(D)** The overlay visualization map of country co-authorship analysis generated by VOSviewer. The node color reflected the corresponding average appearing year (AAY) according to the color gradient in the lower right corner.

Between 1981 and 2024, researchers from 93 countries and regions have contributed to publications on gut microbiota and bariatric surgery. Figure 3A illustrates the geographical distribution of research in this field, showing that studies are primarily concentrated in North America, Asia, and Europe. Table 1 lists the top 10 most active countries, with the United States leading with 825 publications, accounting for 32.45% of the total. Following the United States are China (298 publications, 11.72%), the United Kingdom (190 publications, 7.47%), France (156 publications, 6.14%), and Italy (154 publications, 6.06%). The United States also has the highest total citation count, reaching 23,084 citations. Although China has more publications than the United Kingdom and France, its total and average citation counts are lower than those of the United Kingdom and France. Figure 3B displays the annual increase in publication volume for the top 10 countries from 1981 to 2024. The United States led in publication count until 2022, after which China’s publication count surpassed that of the United States. Figures 3C,D present visualizations of the international research collaboration network, highlighting the strong collaboration between the United States and China. The connection strength between these two countries is significantly higher than that of other countries, indicating a higher level of cooperation in this field. Additionally, the color intensity of the lines in Figure 3D reflects the starting time of research activities in each country. According to the color gradient shown in the lower right corner of the figure, the United States and the United Kingdom were early participants in this field, while China joined later but has shown a significant increase in research collaboration activity in recent years.

**Table 1:** Top 10 active countries.

Rank	Country	NP	Percent	CPP	TC	TLS
1.	USA	825	32.45	52.16	43,031	363
2.	China	298	11.72	21.83	6,506	121
3.	England	190	7.47	55.51	10,546	266
4.	France	156	6.14	69.28	10,808	169
5.	Italy	154	6.06	43.50	6,699	194
6.	Germany	147	5.78	41.94	6,165	184
7.	Spain	114	4.48	27.03	3,081	137
8.	Sweden	100	3.93	108.65	10,865	141
9.	Brazil	98	3.86	19.17	1,879	38
10.	Canada	98	3.86	43.08	4,222	75

### 3.3 Active institutions

Our systematic search revealed that a total of 1,002 institutions have contributed to publications on gut microbiota and bariatric surgery. The University of São Paulo had the highest number of publications (*n* = 40), followed by the University of Michigan and Mayo Clinic, each with 39 publications (Table 2). Among the top 10 active institutions, five are located in the United States, with the remaining institutions based in Brazil, Sweden, Denmark, the United Kingdom, and France. Notably, while the University of Copenhagen ranks fifth in terms of publication count, it holds the highest total and average citation counts.

**Table 2:** Top 10 active institutions.

Rank	Institutions	Countries	NP	TLS	TC	CPP
1.	University of São Paulo	Brazilian	40	28	1,058	26.45
2.	Mayo Clinic	USA	39	111	3,032	77.74
3.	University of Michigan	USA	39	75	1,235	31.67
4.	University of Gothenburg	Sweden	38	96	7,961	209.50
5.	University of Copenhagen	Denmark	37	163	9,942	268.70
6.	Harvard Medical School	USA	34	104	1,007	29.62
7.	Imperial College London	England	31	105	1,180	38.06
8.	Columbia University	USA	26	51	655	25.19
9.	Washington University	USA	25	49	2,114	84.56
10.	Hopitaux Universitaires Pitie Salpetriere	French	24	98	4,128	172.00

**Figure 4:**
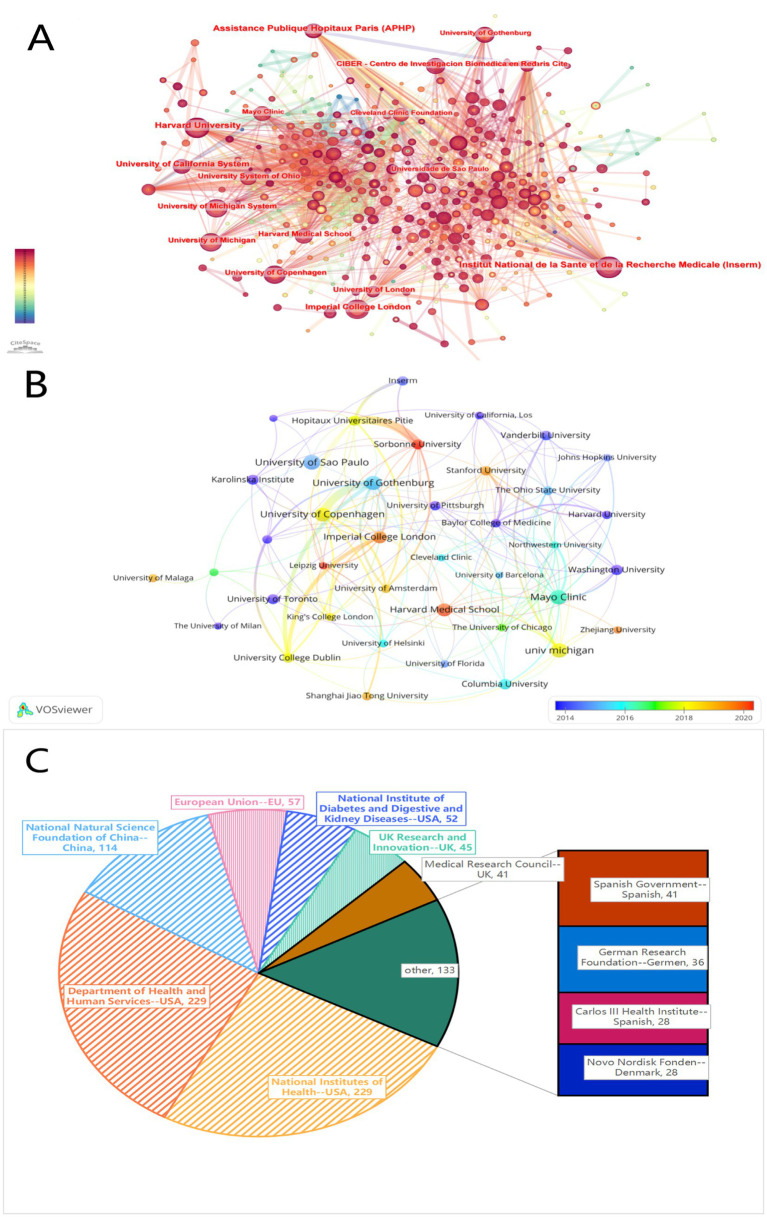
**(A)** The network visualization map of journal co-authorship analysis. **(B)** The overlay visualization map of journal co-authorship analysis. **(C)** The top 10 most active funding agencies involved in this field.

We utilized CiteSpace to create a network visualization map for the co-author analysis of the research (Figure 4A). The nodes represent different institutions, and the colors between the nodes indicate the cooperation between institutions during different periods. A visualization overlay map of the research collaboration network between institutions was created using VOSviewer, encompassing 40 institutions that have published at least 15 studies in this field. Each node in the map represents a research institution, with the size of the node proportional to the number of publications by that institution. The lines between nodes indicate collaborative relationships, with the color variations reflecting different periods of collaboration (Figure 4B). The map shows that institutions such as the University of Copenhagen, Mayo Clinic, Imperial College London, and Harvard Medical School have stronger collaborative relationships with other institutions.

Additionally, we identified the top 10 most active funding agencies in this field (Figure 4C). The U.S. Department of Health and Human Services and the National Institutes of Health lead with the most publications (n = 229 each), followed by the National Natural Science Foundation of China (n = 114) and the National Institute of Diabetes and Digestive and Kidney Diseases (n = 52). The majority of funding in this research area is provided by agencies from the United States and China.

### 3.4 Active journals

**Figure 5:**
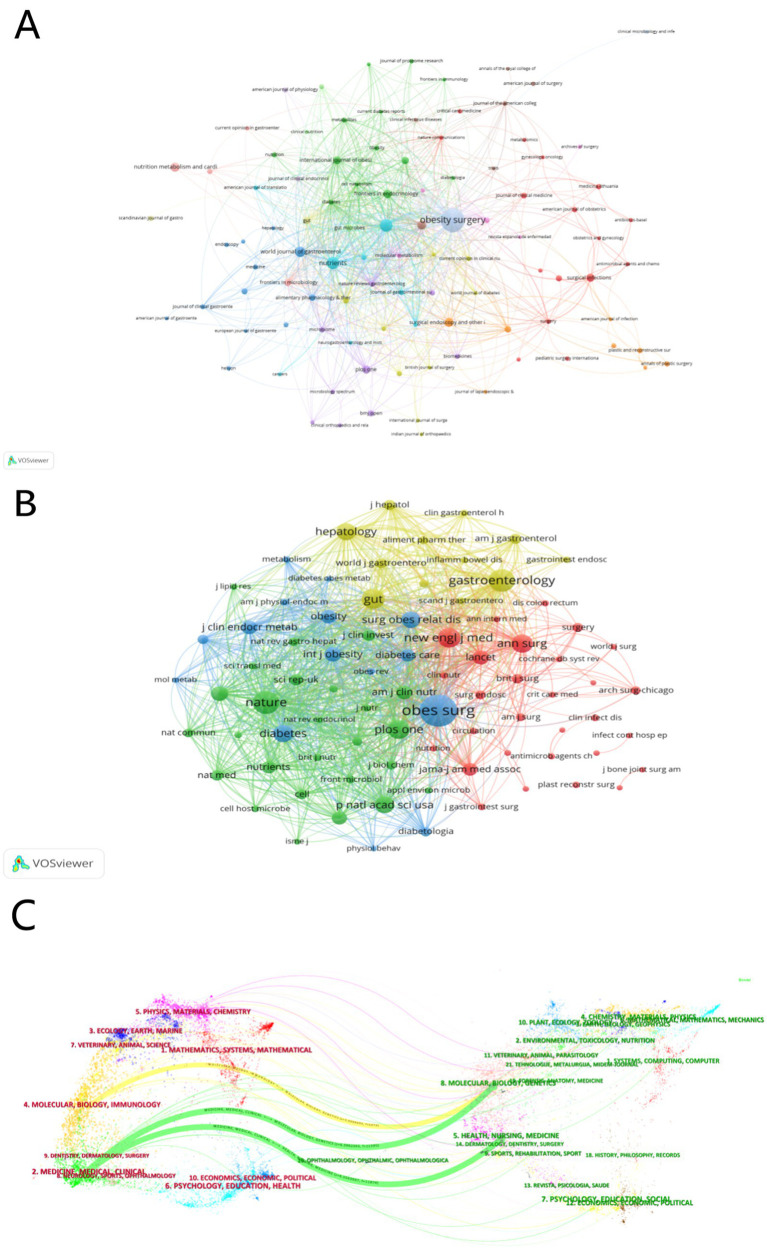
**(A)** The network visualization maps of citing journals. **(B)** The network visualization maps of co-cited journals. **(C)** The dual-map overlay of journals related to BS/GM from 2004 to 2023 by CiteSpace.

Journals serve as key reference points in the academic evaluation system. In specific research areas, journals with the highest number of publications often have significant academic influence, providing researchers with insights into research hotspots and development trends. Our systematic search identified 108 journals that have published literature on gut microbiota and bariatric surgery. Among them, as shown in Table 3, Obesity Surgery has the highest number of publications (n = 178), far exceeding other journals, followed by Surgery for Obesity and Related Diseases (n = 52) and Nutrients (n = 48). Among the top 10 journals in terms of publication volume, Obesity Surgery has the highest total citation count (TC = 3,529). Nutrients have the highest impact factor (4.8), while PLOS ONE has the highest h-index (268). As shown in Figures 5A,B, we used VOSviewer to generate network visualizations of cited and co-cited journals. Obesity Surgery achieved the highest total link strength (TLS) of 765 for co-citations, followed by Surgery for Obesity and Related Diseases (TLS = 398), Nutrients (TLS = 383), and Frontiers in Endocrinology (TLS = 120). Additionally, we used CiteSpace to create a dual-map overlay of journals, illustrating their thematic distribution and citation pathways. The map employs lines of two primary colors to indicate citation and co-citation relationships between journals (Figure 5C).

**Table 3:** Top 10 active journals.

Rank	Journal	Country	NP	TLS	TC	IF	JCR	H-idex
1.	Obesity Surgery	USA	178	765	3,529	2.9	3	128
2.	Surgery for Obesity and Related Diseases	USA	52	398	990	3.5	3	75
3.	Nutrients	Switzerland	48	383	1,264	4.8	2	75
4.	World Journal of Gastroenterology	China	34	91	1,355	4.3	3	129
5.	Nutrition Metabolism and Cardiovascular Diseases	Italy	27	5	534	3.3	3	84
6.	Scientific Reports	England	25	85	381	3.8	2	149
7.	Surgical Endoscopy and Other Interventional Techniques	USA	25	50	660	2.4	2	141
8.	Frontiers in Endocrinology	USA	23	120	290	3.9	2	51
9.	Plos One	USA	20	44	969	2.9	3	268
10.	Surgical Infections	USA	20	43	297	1.4	4	51

### 3.5 Active authors

**Figure 6:**
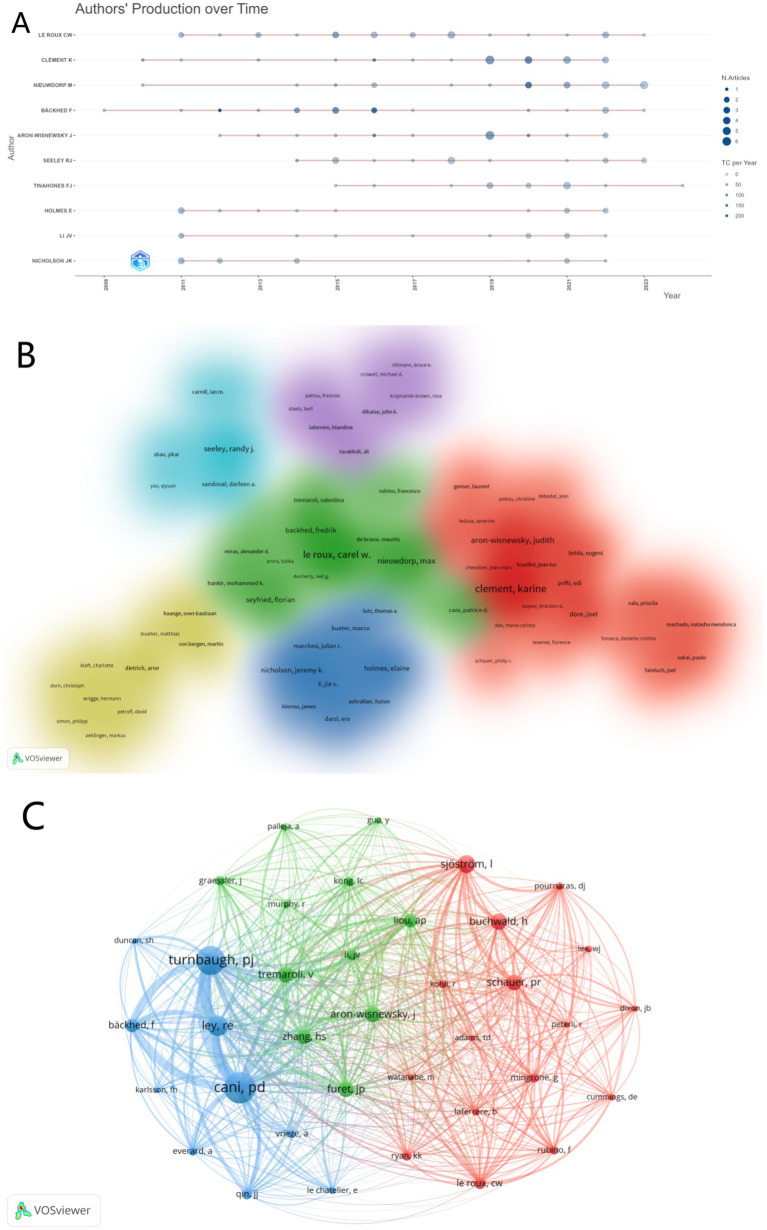
**(A)** The top 10 authors’ production over time. The circle size represented the number of documents, and the shade of the color signified the total number of citations. **(B)** The cluster visualization map of author cooperation analysis. Authors with close collaborative relationships are assigned to the same cluster with the same color. **(C)** The network visualization map of author co-authorship analysis.

From the author publication data, Karine Clement has the highest number of publications, with a total of 24 papers (Table 4). However, Jeremy K. Nicholson has the highest h-index, at 135. Figure 6A shows the dynamic changes in the annual output and citation counts of the top 10 authors from 1981 to 2024. Additionally, we analyzed authors with more than six publications and created a cluster density map of their collaboration networks (Figure 6B). Six distinct clusters of collaboration among these authors naturally emerged. For the co-citation analysis of authors, we used total link strength (TLS) as the measurement metric. Figure 6C depicts 37 authors with citation counts exceeding 100. The top three authors by TLS are Patrice D. Cani (TLS = 6,061), Peter J. Turnbaugh (TLS = 6,019), and Ruth E. Ley (TLS = 4,318).

**Table 4:** Top ten active authors.

Rank	Author	Documents	Citations	TLS	H-idex	CPP
1	Clement, Karine	24	4,327	60	46	180.29
2	Le Roux, Carel	21	1771	38	66	84.33
3	Aron-Wisnewsky, Judith	15	3,236	43	35	215.73
4	Nieuwdorp, Max	15	1786	14	69	119.07
5	Seeley, Randy J.	14	1,199	12	98	85.64
6	Tinahones, Francisco Jose	13	379	33	63	29.15
7	Holmes, Elaine	12	868	43	115	72.33
8	Backhed, Fredrik	11	7,089	15	104	644.45
9	Nicholson, Jeremy K.	11	877	41	135	79.73
10	Seyfried, Florian	11	208	23	20	18.91

### 3.6 Reference and co-citation analysis

**Figure 7:**
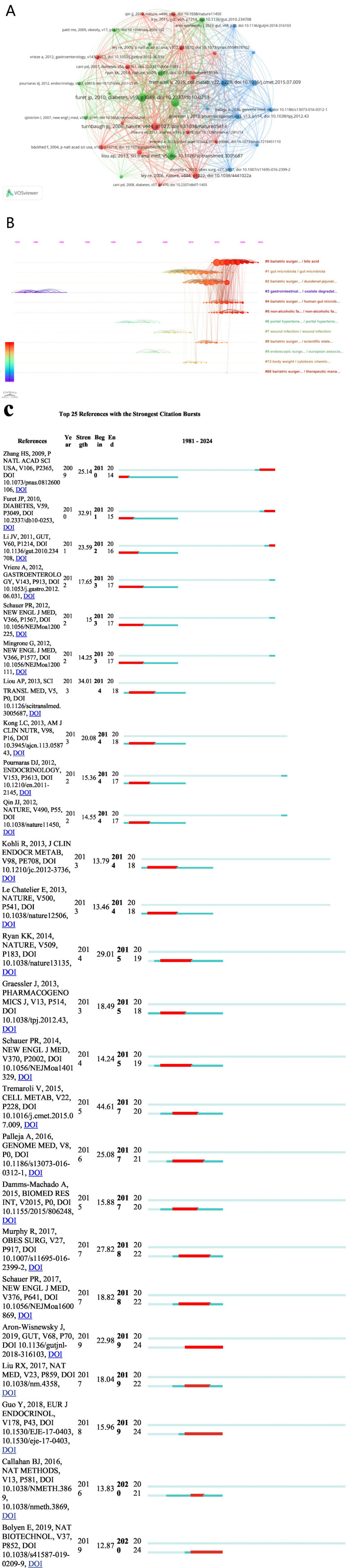
**(A)** The cluster view map of reference co-citation analysis generated by VOSviwer. **(B)** The timeline view map of reference co-citation analysis generated by CiteSpace. **(C)** The top 25 references with the strongest citation bursts generated by CiteSpace. The bars in blue represented the timeline; the bars in red represented a burst period of the references.

We used VOSviewer to generate a co-citation analysis map of references, where larger nodes represent more frequently cited documents, and the lines between nodes indicate mutual citations between documents (Figure 7A). Based on this, we created a timeline of co-citation clusters (Figure 7B), which helps researchers understand the hotspots and trends within the research field. The horizontal axis of the figure represents the timeline, with points on the left indicating earlier citations and points on the right indicating more recent citations.

From the timeline, it is evident that current research focuses include bariatric surgery and bile acids, bariatric surgery and gut microbiota, and non-alcoholic fatty liver disease. Additionally, we used CiteSpace to generate a visualization of the top 25 references with citation bursts (Figure 7C). This figure shows that the first significant burst in citations within this field occurred in 2010, triggered by a paper published by Zhang et al. (2009). The three papers with the highest burst strengths were published by Tremaroli et al. (2015), Liou et al. (2013), and Furet et al. (2010). Their burst strengths were 44.61, 34.01, and 32.91, respectively. The most recent citation burst occurred in 2020, triggered by a paper published by Bolyen et al. (2019).

**Figure 8:**
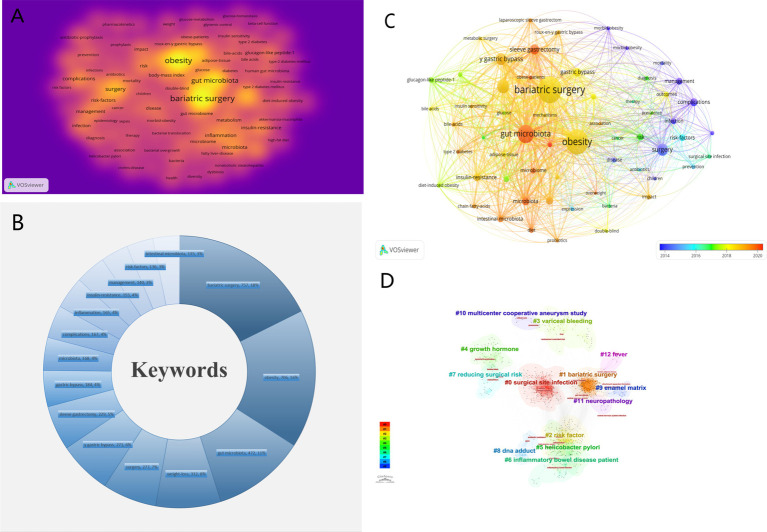
**(A)** The cluster visualization map of keywords analysis. **(B)** The top 15 keywords appear over time. **(C)** The network visualization maps of keywords generated by VOSviwer. **(D)** The cluster view map of keywords analysis generated by CiteSpace.

**Figure 9:**
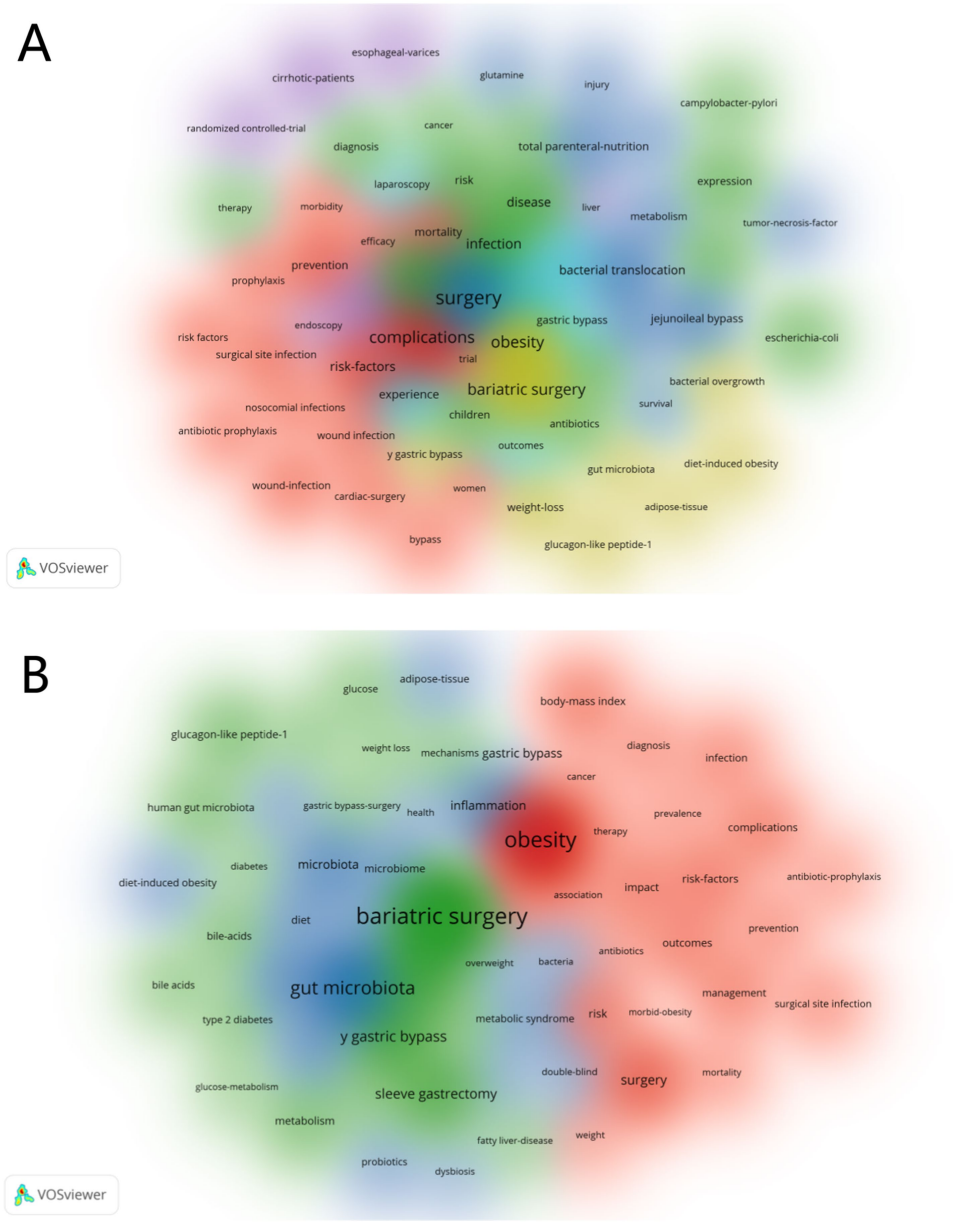
**(A)** The keywords landscape for the period from 2004 to 2012. **(B)** The keywords landscape for the period from 2013 to 2024.

**Figure 10:**
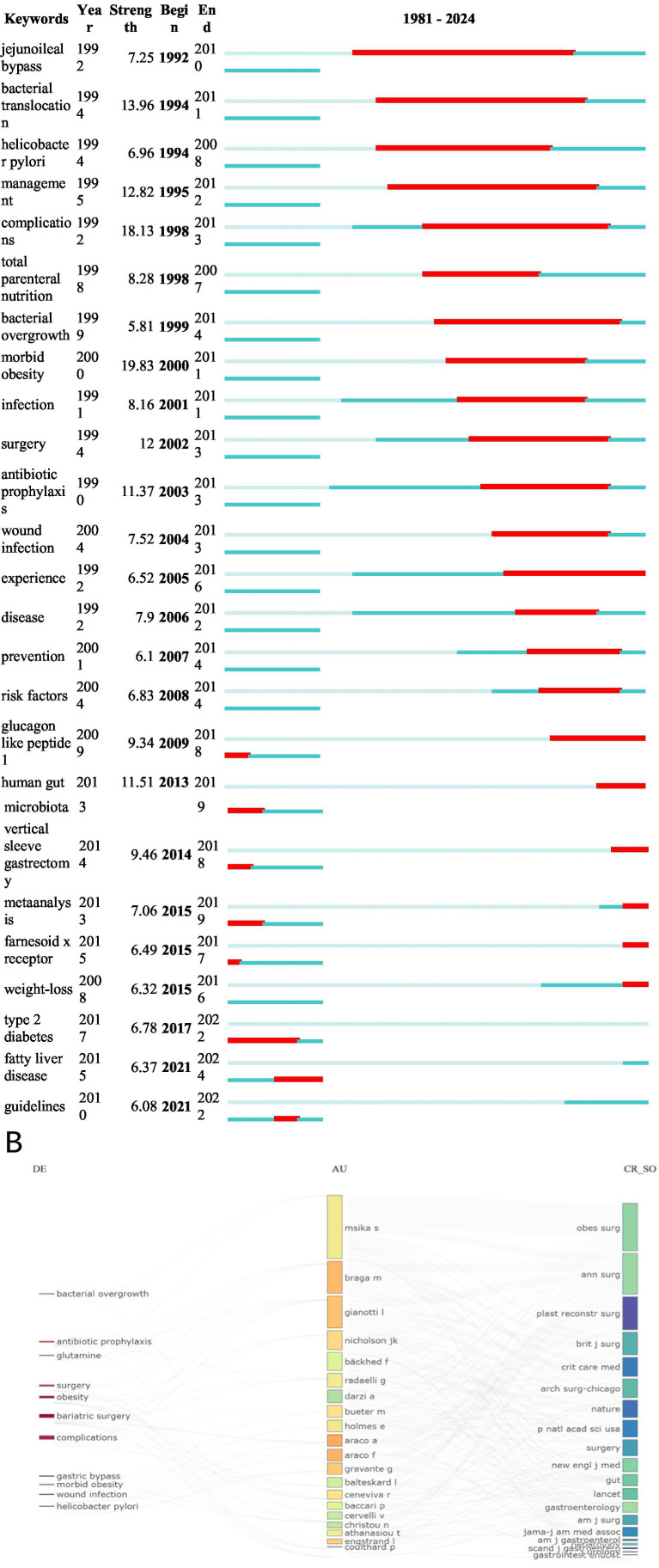
**(A)** The top 25 keywords with the strongest citation bursts generated by CiteSpace. **(B)** Keyword-author-journal correlation map for the field.

We used VOSviewer to generate a visual map of keyword clustering analysis (Figure 8A). Figure 8B shows the top 15 most frequently occurring keywords, with “bariatric surgery,” “obesity,” and “microbiota” being the most frequent. To illustrate the evolution of keywords over time, we created a timeline of keyword occurrences (Figure 8C). Red nodes represent recently frequent keywords, such as “gut microbiota,” “gastric bypass,” and “microbiome,” reflecting current research hotspots. Additionally, we used CiteSpace to generate a keyword clustering analysis map (Figure 8D), which includes 13 clusters: #0 Surgical site infections, #1 Bariatric surgery, #2 Risk factors, #3 Variceal bleeding, #4 Growth hormone, #5 *Helicobacter pylori*, #6 Inflammatory bowel disease patients, #7 Lowering risk factors, #8 DNA adducts, #9 Enamel matrix, #10 Multicenter cooperative aneurysm study, #11 Neuropathology, #12 Fever. Cluster analysis helps quickly identify major themes and sub-themes in research, and assists researchers in discovering gaps in existing studies. Beyond quantitative analysis, this study also conducted a qualitative analysis of keywords. Figures 9A,B show the visualization of high-frequency keywords for the periods 1981–2012 and 2013–2024, respectively. We summarized newly emerging and increasingly frequent keywords during 2012–2024, as listed in Table 5. Figure 10A presents the top 25 most-cited keywords. Furthermore, we created a keyword-author-journal association map (Figure 10B) to help researchers identify key authors and influential journals in the field, as well as their interconnections.

**Table 5:** The qualitative analysis of keywords.

New research keywords in 2012–2024			Increased keywords popularity in 2012–2024		
Mechanism	Treatment	Diagnosis	Mechanism	Treatment	Diagnosis
Antibiotic-prophylaxis, efficacy, trial, complications, bacterial overgrowth, helicobacter-pylori, wound-infection, morbid-obesity, cytokines, infection, esophageal-varices, cirrhotic-patients, cigarette-smoking, mouse model, bacterial-infection, nosocomial infections, cholecystectomy, *Escherichia coli*, sepsis, purification, cancer-patients	Cardiac-surgery, band ligation, abdominal-surgery, enteral nutrition, anesthesia, jejunoileal bypass, gastrointestinal surgery	Surgery, management	Blood-glucose, colorectal-cancer, butyrate, body-weight, nonalcoholic steatohepatitis, fatty liver-disease, glucagon-like peptide-1, plasma ghrelin levels, risk-factors	Mediterranean diet, sleeve gastrectomy, Y gastric bypass	Therapy

## 4 Discussion

Increasing research has demonstrated a close relationship between gut microbiota and bariatric surgery, indicating their significant role in weight reduction (Santos et al., 2021; Luijten et al., 2019; Akagbosu et al., 2022; Han et al., 2022). This study represents the first bibliometric analysis of literature related to bariatric surgery and gut microbiota using the Web of Science Core Collection (WoSCC) database. By statistically analyzing 2,542 publications from the WoSCC database, we created visual maps to examine research trends over the past 40 years and potential future research hotspots, aiming to provide a valuable reference for researchers in this field. The number of publications and their growth trends are crucial indicators of research activity within an academic field. As shown in Figure 2, the number of publications on BS and GM has generally increased over the past 40 years, which can be divided into three phases: Phase 1 (1981–1991): The number of publications was relatively low and stable. Phase 2 (1992–2014): The publication rate gradually accelerated. Phase 3 (2015–2024): The number of publications surged dramatically. This significant growth trend can be attributed to two main factors. First, the increasing global prevalence of obesity and the rising number of patients undergoing bariatric surgery have driven the demand for related research. Second, advancements in 16S rRNA gene sequencing and metagenomic sequencing technologies have provided sophisticated tools for in-depth studies of gut microbiota. Based on the growth trend in publication numbers and the analysis of the aforementioned factors, it is reasonable to infer that this field has become a focal point for researchers in weight loss treatments. The application and mechanisms of gut microbiota in weight loss treatments are rapidly becoming a future research hotspot. Further exploration in this area could offer new approaches for the prevention and treatment of obesity, thereby advancing personalized and precision medicine.

This paper systematically compiles research literature from 95 countries worldwide, revealing that countries with higher publication volumes are primarily concentrated in North America, Asia, and Europe (Figure 3A). Globally, the United States has the highest number of publications (n = 825), accounting for 32.45% of the total, significantly outpacing other countries. The United States also leads in citation counts (Table 1), reflecting its leading position and academic contributions in this research field. Analysis of the trends in publication volumes over time (Figure 3B) indicates that although China entered this research field later, its publication volume has grown significantly in recent years, gradually approaching that of the United States. China is now the second-largest contributor globally and the most prominent research force in Asia. This can be attributed to two main factors: first, the rapid increase in obesity rates in China. According to BMI classifications, 34.8% of Chinese adults are overweight, and 14.1% are obese. Given China’s large population, these percentages correspond to very high absolute numbers (Wang et al., 2021; Chen et al., 2023). Second, the rising obesity rates pose a significant challenge to the public health system, prompting increased national funding for obesity treatment and related research. However, the average citation count of Chinese publications remains relatively low. Regarding international collaboration, the United States has the closest interactions with China and collaborates with the United Kingdom and Canada (Figure 3D). International scientific cooperation is crucial for advancing specific research fields, and fostering a mutually beneficial scenario. By visualizing the involvement of different countries in this research area from various perspectives, we can highlight leading nations and potential partners, promoting broader international research collaborations and further development in this field.

In terms of institutional analysis, the top 10 organizations in publication volume come from the United States, Brazil, Sweden, Denmark, the United Kingdom, and France (Table 2). Institutions from the United States account for 50% of the top 10, reflecting the country’s strong research capabilities in this field. The publication volumes of the top five institutions are very close, with the University of São Paulo having the highest number of publications. Although the University of Copenhagen ranks fifth in total publications, it leads in both total citations and average citations per paper, likely due to its extensive research collaborations. The overlay visualization of the inter-institutional research collaboration network (Figure 4B) shows that the University of Copenhagen collaborates most frequently with other institutions. However, Sorbonne University and Leipzig University have also strengthened their collaborations with other institutions in recent years, possibly because they started contributing to this research field relatively late. Among the top 10 funding agencies, institutions from the United States again dominate, accounting for 50% (Figure 4C). Adequate financial support is crucial for ongoing progress in research, which may be one of the primary reasons the United States maintains a leading position in this field. In summary, through multidimensional visual analysis of institutions from various countries, we can identify which institutions are at the forefront of this research area and understand the collaboration relationships between them. This information helps researchers find suitable partners in this field, further promoting inter-institutional cooperation.

Table 4 summarizes the main information about the top 10 journals in terms of publication volume in the field of bariatric surgery and gut microbiota. The journal Obesity Surgery has the highest number of publications, followed by Surgery for Obesity and Related Diseases and Nutrients, which are classified as Q3 and Q2 in the Journal Citation Reports (JCR), respectively. In terms of co-citations, Obesity Surgery, Surgery for Obesity and Related Diseases, and Nutrients are also the top three journals (Figures 5A,B), indicating their high academic standing in this field. The dual-map overlay of journals (Figure 5C) shows that research published in journals categorized under molecular/biology/genetics or health/nursing/medicine is often cited in molecular/biology/immunology and medicine/clinical journals. Journal analysis plays a crucial role in bibliometrics. By combining quantitative and qualitative research methods, we assessed the academic impact and quality levels of the major journals in this field. This provides a scientific basis for researchers to select journals, helping them identify and choose high-quality journals that match their research areas.

Through co-authorship analysis and Table 5, it is evident that the most prolific author is Karine Clement from Sorbonne University. She has long been dedicated to research in nutrition and gastroenterology. Her most cited paper is “Gut Microbiota and human NAFLD: disentangling microbial signatures from metabolic disorders,” published in 2020. This study utilized various gut microbiota sequencing tools and NAFLD diagnostic methods to provide new insights into different microbiome characteristics (Aron-Wisnewsky et al., 2020). In 2019, she published “Major microbiota dysbiosis in severe obesity: fate after bariatric surgery,” which confirmed significant changes in the gut microbiota due to severe obesity, including a reduction in microbial genetic resources (MGR) and functional pathways associated with metabolic deterioration (Aron-Wisnewsky et al., 2019). Additional strategies are needed post-BS to fully restore these changes and improve the gut ecosystem and microbiome-host interactions in severely obese patients. The second most prolific author is Professor Carel W. le Roux from the Diabetes Complications Research Centre at UCD Conway Institute, University College Dublin. In 2013, he published a study on the mechanisms of weight loss after bariatric surgery, indicating that changes in the gut microbiota are a significant cause of weight loss rather than a result (Sánchez-Alcoholado et al., 2019). In 2015, he investigated the impact of post-bariatric surgery gut microbiota changes on fat regulation by transplanting the gut microbiota of bariatric surgery patients into germ-free mice. He found that the altered microbiota post-surgery promoted reduced fat deposition in recipient mice, concluding that the gut microbiota plays a direct role in weight reduction post-surgery (Morales-Marroquin et al., 2020). This paper has been cited 589 times. Although Professor Fredrik Bäckhed ranks eighth in publication volume, he has the highest average and total citation counts. He contends that targeting the gut microbiota presents substantial potential for advancing human health. The development of next-generation probiotics is identified as a promising strategy for modulating the gut microbiome and enhancing human health. This is consistent with the future research hotspots we have analyzed (Khan et al., 2023). When evaluating a researcher’s academic contributions in a specific field, it is essential not only to consider the number of published papers but also the citation counts and the quality of the journals in which they are published. These metrics indicate that the aforementioned authors have made significant contributions to the field and suggest that they are likely to achieve further advancements in the future.

Citation analysis can evaluate the actual academic impact and current research hotspots in a specific field. The paper by Zhang H. et al., published in 2009 in the Proceedings of the National Academy of Sciences of the United States of America, is the earliest and most cited article in this field. This study observed that Roux-en-Y gastric bypass surgery significantly altered fecal microbiota composition, leading to an increase in *γ*-Proteobacteria (96.2% of which were Enterobacteriaceae members) and a decrease in Firmicutes and methanogens. These changes likely reflect the dual impact of surgery-induced alterations in gut microbiota and subsequent changes in food intake and digestion (Zhang et al., 2009). Additionally, a study by Judith et al., published in 2019, reported the highest burst strength (Aron-Wisnewsky et al., 2019). This comprehensive study on severely obese patients found that 75% of these patients had reduced gut microbiota gene richness (MGR), closely associated with significant metabolic complications. The research suggested that reduced MGR might be a critical factor leading to severe obesity. Although bariatric surgery can improve MGR and achieve weight loss in the short term, most patients continue to exhibit low MGR levels long after surgery. Investigating whether drugs can improve MGR is a future research direction and goal. Through a comprehensive analysis of keywords, we identified the current research frontiers and hotspots in three main areas: 1. Differences and mechanisms in GM diversity and richness changes after different bariatric surgery procedures. 2. Mechanisms of GM in weight loss treatments. 3. Application of GM-based combination therapies in the weight loss field.

Studies have shown that bariatric surgery alters the gut microbiota, and different surgical procedures have varying effects on the gut microbial community. Sleeve gastrectomy (SG), Roux-en-Y gastric bypass (RYGB), and adjustable gastric banding (AGB) each result in distinct post-operative microbiota compositions and abundances, with RYGB causing the most significant changes. These changes may be related to adaptive adjustments in the gut environment or fluctuations in pH levels (Santos et al., 2021; Morales-Marroquin et al., 2020; Khan et al., 2023; Ilhan et al., 2017). Although many studies have explored the changes in gut microbiota before and after bariatric surgery, multiple post-operative factors—including dietary habits, medication use, infection status, and circadian rhythms—may influence the detection of gut microbiota. Therefore, further in-depth research is needed to analyze the changes in gut microbiota after bariatric surgery. Changes in the composition of the gut microbiota are associated with obesity and can even vary with the degree of obesity. Certain obesity-related gut microbiota have a heightened ability to extract energy from the diet, which can lead to increased energy intake (Musso et al., 2011). Research shows that continuous feeding of the B. hetaiotaomicron strain to mice can reduce the concentration of glutamate in the blood and mitigate weight gain and obesity caused by a high-fat diet (Liu et al., 2017). An increase in the ratio of Firmicutes to Bacteroidetes is associated with promoting obesity (Ley et al., 2005). In obese individuals, the composition of genera such as Akkermansia, Faecalibacterium, Oscillibacter, and Alistipes is significantly reduced, while the proportion of *Lactobacillus reuteri* is relatively higher (Thingholm et al., 2019). This may be related to the different metabolites produced by various gut bacterial genera through their specific metabolic pathways, which can lead to alterations in metabolic processes. Additionally, the gut microbiota can influence metabolism through inflammatory responses, immune regulation, and the gut-brain axis (Boulangé et al., 2016; Li et al., 2018; Hartstra et al., 2015; Geng et al., 2022). Given these findings, it is crucial to further investigate the relationship between bariatric surgery and the gut microbiota. In the future, microbial preparations could potentially be used to regulate the composition and function of the gut microbiota, thereby influencing metabolism and achieving weight loss goals.

This study has several strengths. It is the first to conduct a comprehensive bibliometric analysis of the research field of bariatric surgery (BS) and gut microbiota (GM) based on the Web of Science Core Collection (WoSCC), filling a research gap and providing a reference for future studies. Utilizing five tools (Microsoft Excel 2021, CiteSpace, VOSviewer, the R package “bibliometrix,” and an online bibliometric analysis platform), we thoroughly and deeply explored and presented the research data (Aria and Cuccurullo, 2017). However, this study also has some limitations. Firstly, the search was conducted solely in the WoSCC database, though the WoSCC is recognized for its high-quality and comprehensive data and is the most commonly used tool in bibliometric analyses. However, research published in other databases (such as Scopus, PubMed, Embase, etc.) may also be significant, and there is a risk of missing relevant studies from alternative databases. Secondly, some non-English studies may have been overlooked due to language restrictions. Lastly, despite our efforts to develop a detailed and comprehensive search strategy, the diversity of keywords may have led to some omissions.

## 5 Conclusion

In summary, this study employed various tools including Excel 2021, VOSviewer 1.6.19, CiteSpace 6.2.R3, the “bibliometrix” package in R 4.3.0, and online bibliometric analysis platforms to conduct a bibliometric and visualization analysis of the fields of BS and GM. Over the past 40 years, research interest in this area has consistently increased. Understanding the role of gut microbiota in metabolism continues to evolve, with an increasing number of studies focusing on the changes in gut microbiota following bariatric surgery and their important roles and mechanisms in weight loss treatment. This study comprehensively analyzed global research advancements in BS and GM, and predicted future research hotspots, including specific changes in GM post-BS and their biological mechanisms, the role of GM in weight loss treatments, Research and application of gut microbiota-based microbiome products in combination with weight loss treatment is a hot topic for future studies. In recent years, the global obesity rate has been rising exponentially, leading to an increasing number of individuals requiring weight loss treatment. Future studies on bariatric surgery (BS) and gut microbiota may aim to achieve non-invasive weight loss solutions, thereby reducing patients’ surgical pain. This article aims to advance this field towards more precise and personalized treatment approaches, providing scientific evidence and new therapeutic methods for the treatment of obesity and its related metabolic diseases.

## Footnotes

^1^ https://bibliometric.com/app
